# Facile Fabrication of 1-Methylimidazole/Cu Nanozyme with Enhanced Laccase Activity for Fast Degradation and Sensitive Detection of Phenol Compounds

**DOI:** 10.3390/molecules27154712

**Published:** 2022-07-23

**Authors:** Yu Lei, Bin He, Shujun Huang, Xinyan Chen, Jian Sun

**Affiliations:** 1 Key Laboratory of Molecular Medicine and Biotherapy in the Ministry of Industry and Information Technology, School of Life Science, Beijing Institute of Technology, Beijing 100081, China; m15810967956@163.com (Y.L.); bhe_bit@163.com (B.H.); 3220191213@bit.edu.cn (S.H.); xychenacad@126.com (X.C.); 2 Advanced Research Institute of Multidisciplinary Science, Beijing Institute of Technology, Beijing 100081, China

**Keywords:** laccase, phenolic oxidation, nanozyme, intelligent detection, self-assembly synthesis

## Abstract

Facile construction of functional nanomaterials with laccase-like activity is important in sustainable chemistry since laccase is featured as an efficient and promising catalyst especially for phenolic degradation but still has the challenges of high cost, low activity, poor stability and unsatisfied recyclability. In this paper, we report a simple method to synthesize nanozymes with enhanced laccase-like activity by the self-assembly of copper ions with various imidazole derivatives. In the case of 1-methylimidazole as the ligand, the as-synthesized nanozyme (denoted as Cu-MIM) has the highest yield and best activity among the nanozymes prepared. Compared to laccase, the *K*_m_ of Cu-MIM nanozyme to phenol is much lower, and the *v*_max_ is 6.8 times higher. In addition, Cu-MIM maintains excellent stability in a variety of harsh environments, such as high pH, high temperature, high salt concentration, organic solvents and long-term storage. Based on the Cu-MIM nanozyme, we established a method for quantitatively detecting phenol concentration through a smartphone, which is believed to have important applications in environmental protection, pollutant detection and other fields.

## 1. Introduction

Natural enzymes are biocatalysts that can efficiently catalyze many reactions under a mild condition. Because of their high substrate specificity and catalytic activity, enzymes are used in biosensors, pharmaceutical processes, the food industry and agriculture [1,2,3]. Although natural enzymes are widely used, there are some shortcomings to be solved towards the practical applications, such as high cost of separation and purification, low thermal/salty stability, unsatisfied environmental tolerance and difficult recyclability [4,5,6]. In order to overcome these limitations of natural enzymes, researchers have developed several strategies including enzyme engineering, enzyme immobilization, enzyme nanoreactors and nanozymes with enzyme-like activities [7,8,9,10]. Enzyme engineering allows enzymes to achieve higher reaction rates and stability, but mutating protein sequences remains a complex task [11]; enzyme immobilization and enzyme nanoreactors improve enzyme stability and reusability, but may also lead to enzyme activity decrease [12]. Among these methods, nanozymes have gained much attention due to their advantages of high designability and structural stability, low cost, mass production and long-term storage [6]. Since Fe_3_O_4_ nanoparticles were found to have peroxidase-like catalytic activity in 2007 [13], many researches have been devoted to the development and application of nanozymes [14]. So far, a variety of nanomaterials such as metal-based [15,16,17], metal oxide-based [18,19,20], carbon-based [21,22,23] and metal-organic frameworks (MOFs) [24,25,26] have been found to have various types of enzymatic catalytic activities including peroxidase, oxidase, catalase, superoxide dismutase, hydrolase and glucose oxidase [27]. These nanozymes have played a good role in biosensors, imaging, cancer treatment, wound healing, antibacterial mechanisms and environmental protection [14,28].

On the other hand, phenol and its derivatives are environmental pollutants with severe toxicity on the human body, animals and plants [29]. Most of the phenolic compounds in the environment are derived from pulp mills, coal mines, refineries, wood preservation factories and various chemical industries [30,31]. Many methods have been tested for phenolic compounds treatment or degradation including photocatalysis [32], biocatalysis [33], nanozyme [34], thermal catalysis [35] and adsorption [36]. Although natural laccase can be used as a green biocatalyst for sewage treatment and soil remediation [37,38], it has some disadvantages including high cost, easy inactivation and poor stability, which seriously limit the practical application [39]. Recently, nanozymes with laccase-like activity have been used in phenolic pollutant degradation. For instance, the composite of Cu/His-Cys formed by the assembly of copper with histidine and lysine was proved to have good laccase-like activity [40], Cu-GMP (Cu-guanosine monophosphate) with non-static MOF structure catalyzes the oxidation of phenolic species faster than laccase [5], Cu_2_O nanoparticles with laccase-like activity exhibit good cycling ability [34] and I-Cu (imidazole-Cu) [41] with the structure of open vesicles exhibits laccase-like activity and strong stability. Despite the fact that these nanozymes have good laccase mimetic properties, they have the disadvantages of expensive raw materials (such as dipeptides), general activity and irregular shapes.

As known, laccase is a multicopper oxidase that contains four Cu ions, one type 1 (T1), one type 2 (T2) and a coupled binuclear type 3 Cu pair (T3). The T2 and T3 centers form a trinuclear Cu cluster that is the active site for O_2_ reduction to H_2_O and each Cu is attached to an imidazole side chain of histidine [42]. In addition, imidazole and its derivatives are good ligands for metal complexes and metalloenzymes [43]. Inspired by the nature of laccase and availability of imidazole, we hypothesize a novel nanozyme fabrication by selecting copper ions and imidazole derivatives with various carbon chain lengths to achieve enhanced laccase-like activity.

Herein, we propose a novel and simple self-assembly strategy for a constructing laccase-like nanozyme based on the structural characteristics of the laccase active site. We used cheap ligands such as imidazole, 1-methylimidazole, 1-ethylimidazole, 1-propylimidazole and 1-butylimidazole to prepare five novel nanozymes, denoted as Cu-IM, Cu-MIM, Cu-EIM, Cu-PIM and Cu-BIM, respectively. Screened Cu-MIM with C1 chain has the best catalytic performance. Compared with laccase, Cu-MIM has a stronger catalytic performance with a MOF-like structure and reaction rate, and also shows high stability in various complex environments. Moreover, we established a smartphone colorimetric assay based on the Cu-MIM nanozyme for fast, convenient and accurate quantitative detection of phenol.

## 2. Results and Discussion

### 2.1. Structural Characterization of Nanozymes

**Figure 1:**
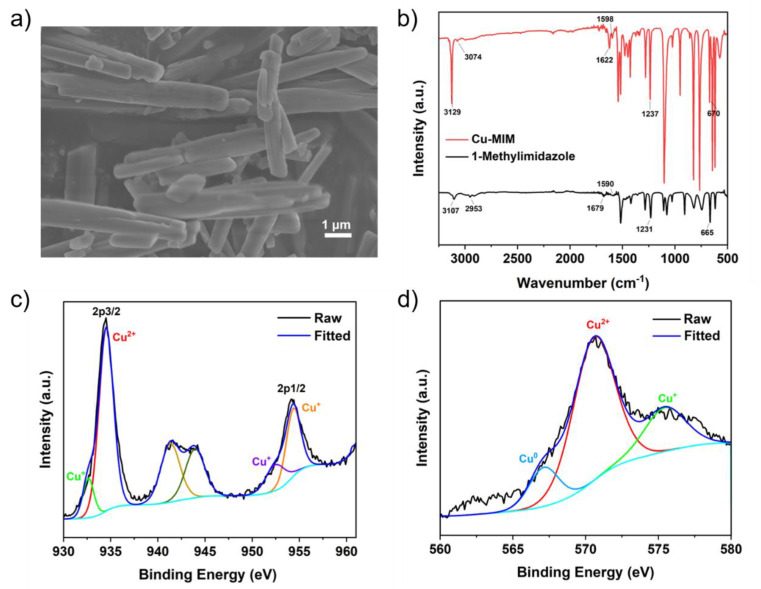
The Cu-MIM nanozyme: (**a**) SEM image; (**b**) FTIR spectrum and one for 1-methylimidazole; (**c**) Cu 2p XPS spectrum; (**d**) Cu LMM Auger spectrum.

As shown in Figure 1a, the Cu-MIM nanozyme has a morphology of nanorods with a diameter of approximately 600 nm and a length of several micrometers. Cu-IM nanozyme has a morphology of cuboid with a short length. Cu-EIM nanozyme shows a nanosheet morphology with a length and width of several microns, while Cu-PIM and Cu-BIM show amorphous structure (Figure S1, ESI). In order to further study the crystal structure of the nanozymes, XRD characterization was performed. As shown in Figure S2, the XRD patterns of these nanozymes indicate that they may have regular crystal structures. This may be attributed to the ordered coordination of copper and imidazoles. The specific surface areas of Cu-IM, Cu-MIM, Cu-EIM, Cu-PIM and Cu-BIM are: 3.9823 m^2^/g, 4.5089 m^2^/g, 0.8801 m^2^/g, 0.8760 m^2^/g and 0.7407 m^2^/g, respectively, indicating that they have almost no pore structure. The copper contents in Cu-IM, Cu-MIM, Cu-EIM, Cu-PIM and Cu-BIM measured by ICP-OES are: 35.22 wt%, 29.46 wt%, 23.79 wt%, 23.34 wt% and 30.36 wt%, respectively.

FTIR spectra of the Cu-MIM nanozyme and 1-methylimidazole are shown in Figure 1b. The characteristic peaks at 3129 cm^−1^ and 3107 cm^−1^ are ascribed to the stretching vibrations of the Cu-MIM nanozyme and 1-methylimidazole, respectively, indicating the existence of -C=C-H bonds in the nanozyme. Stretching vibrations at 1622 cm^−1^ and 1679 cm^−1^, and at 1598 cm^−1^ and 1590 cm^−1^, indicate the existence of C=N and C=C, respectively. The stretching vibrations at 1237 cm^−1^ and 1231 cm^−1^ indicate the existence of N-C, and the flexural vibrations at 670 cm^−1^ and 665 cm^−1^ indicate the existence of C-H. These results confirm that 1-methylimidazole exists in the Cu-MIM nanozyme. The positions of characteristic peaks of the Cu-MIM nanozyme and 1-methylimidazole are slightly different, which may be caused by the coordination of Cu ions with N atoms in the imidazole rings.

**Figure 2:**
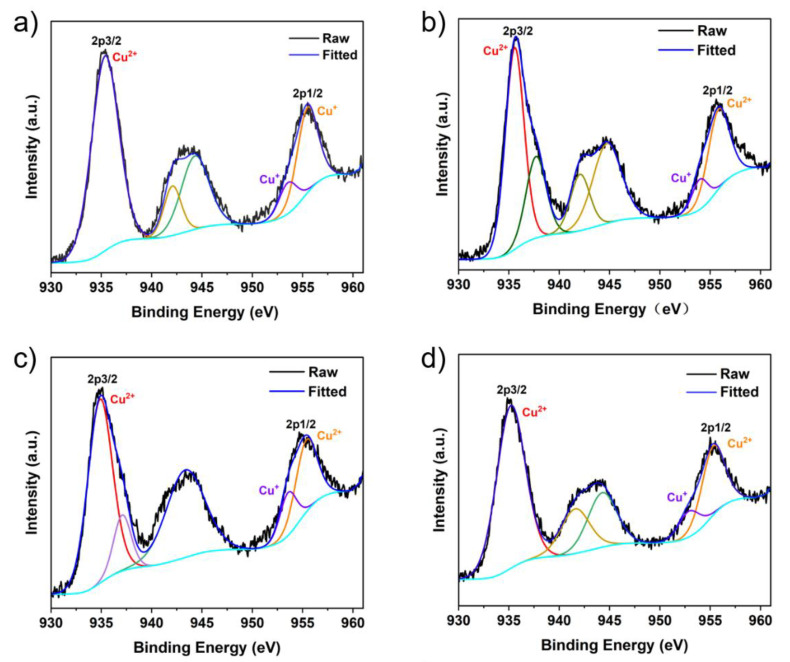
Cu 2p XPS spectrum: (**a**) Cu-IM, (**b**) Cu-EIM, (**c**) Cu-PIM, (**d**) Cu-BIM.

The valence state of Cu in the nanozymes was analyzed by XPS. In Figure S4a, full scan spectrum shows Cu, N, C, O elements in the Cu-MIM nanozyme, which are present in 1-methylimidazole and CuCl_2_. The peaks at 934.52 and 954.36 eV in the high-resolution XPS spectrum (Figure 1c) are assigned to the Cu 2p3/2 and Cu 2p1/2 electrons of Cu^2+^, respectively [44,45]. The lower binding energy peaks at 932.63 (2p3/2) and 952.44 eV (2p1/2) indicate the presence of Cu^+^. For a more comprehensive analysis of the valence state of copper ions in the Cu-MIM nanozyme, the Cu LMM Auger spectrum is shown in Figure 1d. The peak at 570.56 eV indicates the presence of Cu^2+^, the peak at 575.44 eV indicates the presence of Cu^+^ and the peak at 567.12 eV indicates the presence of Cu^0^ in the Cu-MIM nanozyme [44,46]. The production of Cu^+^ and Cu^0^ may be due to the reduction of Cu^2+^ by ethanol during the synthesis process. In addition, the contents of Cu^2+^, Cu^+^ and Cu^0^ in the Cu-MIM nanozyme are 65%, 21% and 14%, respectively. As shown in Figure 2, the other four imitating enzymes only have Cu^2+^ peaks, and Cu^+^ peaks are almost absent. Containing more Cu^+^ may be the reason for the better activity of the Cu-MIM nanozyme. To study the binding of copper and imidazole, the XPS studies of nitrogen (Figure S5) were carried out. The XPS of the N showed two binding energy values at ~399 and ~400 eV. The values are assigned to the free N (-N) of the imidazole group and the coordinated N (=N) with Cu [47]. According to the shift in the FTIR spectral peak positions, Cu may coordinate with -N too.

### 2.2. Catalytic Performance of the Cu-MIM Nanozyme

According to the method described in Section 3.2, five nanozymes (i.e., Cu-IM, Cu-MIM, Cu-EIM, Cu-PIM and Cu-BIM) were synthesized, and their yields were 68.1%, 90.6%, 48.8%, 43.7% and 27.4%, respectively. We found that the yield of Cu-MIM was the highest and the yield of the nanozymes showed a decreasing trend with the increase in the carbon chain length in the imidazole ring. This may be because a long length of the carbon chain increases the steric hindrance, making it difficult for copper to bind to the ligand. Compared with Cu-IM without the carbon chain, the yield of Cu-MIM is still higher, which indicates that the C1 chain can promote the coordination between copper and imidazole to a certain extent.

**Figure 3:**
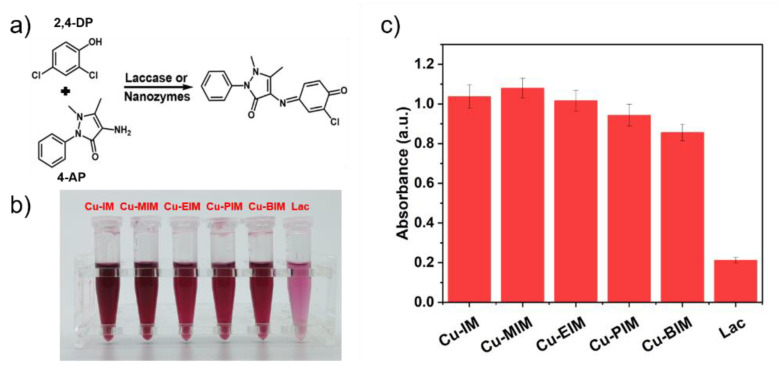
(**a**) Oxidative reactions of 2,4-DP and 4-AP by laccases or nanozymes; (**b**) Picture of 2,4-DP and 4-AP catalyzed by laccase and nanozymes; (**c**) Absorbances at 335 nm of the catalytic products of laccase or nanozymes.

The catalytic activities of laccases and nanozymes were measured by the chromogenic reaction of 2,4-DP and 4-AP for 20 min (Figure S6b) [5]. Compared with the reaction time of 1 h for nanozymes in other works [5,40], Cu-MIM can be catalyzed in 20 min, which greatly shortens the reaction time and shows that Cu-MIM has excellent catalytic activity. The role of 4-AP is to couple with the product to generate a red product, which has a maximum absorption at 335 nm (Figure S6a). As shown in Figure 3a, the products catalyzed by laccase are light red, but those results catalyzed by all the nanozymes are dark red, indicating that the catalytic activity of the synthesized nanozymes is better than laccase by naked eye observation. By comparing the absorbance values after the redox reaction, the most intuitive activities of the five nanozymes were obtained. Each nanozyme is 4–5 times more active than laccase and the Cu-MIM nanozyme performed the best, 5 times that of laccase, as shown in Figure 3c. In Section 2.1, the copper content and specific surface area of each nanozyme have been obtained, and the activity size of each copper site in the nanozyme can be calculated by normalizing the copper content per unit area. Assuming that the copper in each nanozyme is uniformly distributed on the surface, the relative activities of each copper atom in Cu-IM, Cu-MIM, Cu-EIM, Cu-PIM and Cu-BIM were calculated from the absorbance values as: 3.1 × 10^−18^, 3.9 × 10^−18^, 4.5 × 10^−18^, 4.2 × 10^−18^, 3.0 × 10^−18^. In this case, the activity sequence of the nanozyme is Cu-EIM > Cu-PIM > Cu-MIM > Cu-IM > Cu-BIM, indicating that Cu-EIM has the best single-site catalytic activity. Due to the different Hammett σ_m_ values of various substituents, the catalytic activities of the corresponding nanozymes are also different [48]. Different comparison methods screened out that Cu-MIM and Cu-EIM have better laccase mimetic properties. Considering the aspects of raw material cost, catalyst yield, intuitive activity and single-site activity, the Cu-MIM nanozyme was selected as the research focus in this work.

**Figure 4:**
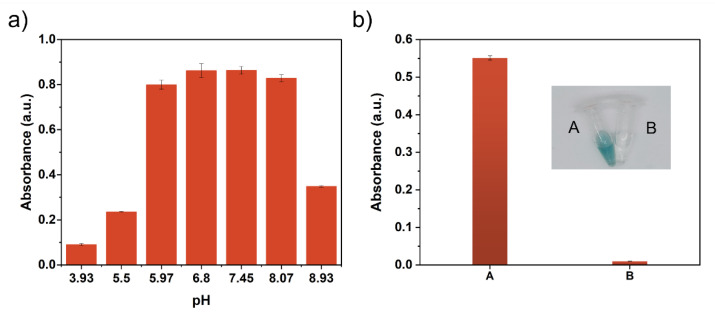
(**a**) pH-dependent activity of the Cu-MIM nanozyme by detecting the absorbance of product at 335 nm; (**b**) Photo of H_2_O_2_ control experiment of the Cu-MIM nanozyme and absorbance at 414 nm; (A) Reaction system containing H_2_O_2_; (B) Reaction system without H_2_O_2_.

As shown in Figure 4a, the Cu-MIM nanozyme shows different activity for the reaction of 2,4-DP and 4-AP reaction with the change in pH value, and comparable activities are achieved when the pH value varies from around 6 to 8. Since laccase also has good catalytic activity under this condition, we use pH 6.8 MES buffer as the reaction media. Figure S6b shows the absorbance curves of products versus time in the systems of laccase and Cu-MIM nanozyme, respectively. The reaction catalyzed by the Cu-MIM nanozyme can be completed within 20 min, indicating a fast detection and degradation performance, while the reaction catalyzed by laccase is carried out slowly and does not reach an equilibrium after 1 h.

Because Cu^2+^ also have a certain catalytic activity, in order to compare the catalytic ability of CuCl_2_ and the Cu-MIM nanozyme, a control experiment was carried out, and the results are shown in Figure S6c. From the color difference, the Cu-MIM nanozyme shows better activity than Cu^2+^.

To further confirm that the activity of Cu-MIM is independent of 1-methylimidazole ligands, comparative experiments were performed. As shown in Figure S6d, the system with only 1-methylimidazole added showed no color change, indicating that it has no catalytic activity for 2,4-DP.

Laccase is an oxidase that oxidizes O_2_ to H_2_O without producing H_2_O_2_, while some oxidations such as glucose oxidase produce H_2_O_2_ in the reaction. In order to verify that the Cu-MIM nanozyme is a laccase-like mimetic enzyme, a control experiment was performed. ABTS and horseradish peroxidase (HRP) were added to the reaction supernatant of the Cu-MIM nanozyme with 2,4-DP. As shown in Figure 4b, there is no color change in sample B, but sample A with H_2_O_2_ added shows a distinct green color. Such results indicate that the Cu-MIM nanozyme does not generate H_2_O_2_ during the catalytic process, which is laccase-like mimetic.

Next, reaction kinetics of laccase and Cu-MIM systems were measured, respectively, at different substrate concentrations (Figure S8). The *K*_m_ and *v*_max_ values are calculated by the Michaelis–Menten model, and the results are shown in Table 1. The *K*_m_ values of the Cu-MIM nanozyme and laccase are both relatively small, indicating that both of them have strong affinity to 2,4-DP. The *v*_max_ of Cu-MIM is 8.5 times higher than that of laccase, indicating that Cu-MIM has an extremely fast reaction rate. This result is still very good compared to other works (Table 1).

The amount of copper in laccase is 0.32 wt% and that in the Cu-MIM nanozyme is 29.46 wt%. When normalizing the amount of copper, it is clear that laccase is more active. However, at the same mass concentration, Cu-MIM has an absolute advantage in activity, and the cost per gram of Cu-MIM raw material (e.g., 1-methylimidazole and copper chloride) is almost negligible compared with the laccase used in this paper. Therefore, Cu-MIM can be used as a very good laccase mimetic.

### 2.3. Catalytic Stability of the Cu-MIM Nanozyme

In practical applications, enzymes are required to maintain high activity and stability under various conditions. Some researchers immobilize laccase in porous carriers to improve its stability [49,50]. To test the stability of the Cu-MIM nanozyme, we compared it with laccase in various harsh environments (different pH incubation, temperature, storage time, ionic strength, ethanol concentration).

**Figure 5:**
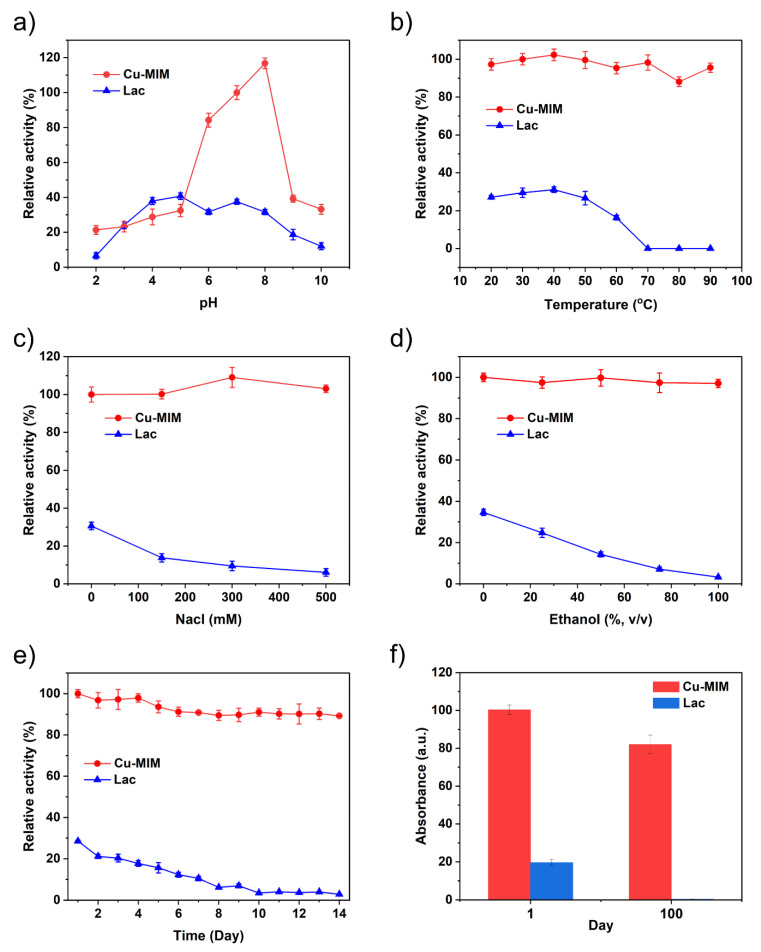
Stability of Cu-MIM and laccase with the same mass concentration at different (**a**) pH, (**b**) temperature, (**c**) NaCl concentration, (**d**) content of ethanol, (**e**,**f**) storage time.

Specifically, we incubated the Cu-MIM nanozyme and laccase in a pH environment of 2–10 for 8 h, and then carried out the catalysis experiments according to the steps described in Section 3.4. As shown in Figure 5a, the catalytic activity of laccase decreases by about 80% after incubation in strongly acidic and basic conditions. Cu-MIM nanozyme shows comparable performances after incubation under strong acidic conditions. Under neutral and basic conditions, the catalytic activity of the Cu-MIM nanozyme is better than that of laccase. Especially under neutral conditions, the Cu-MIM nanozyme shows 3–4 times maximal activity than laccase. The results confirm that Cu-MIM has better pH stability.

In order to study the thermal stability of the Cu-MIM nanozyme, we stored it and laccase at 20−90 °C for 30 min, respectively, and then measured the activity in pH 6.8 MES buffer. As shown in Figure 5b, the activity of laccase increases slightly before 40 °C, then decreases rapidly with further increasing temperature until inactivated at 70 °C. Cu-MIM nanozyme shows excellent thermal stability and maintained good catalytic activity even at a high temperature of 90 °C, with the catalytic activity only decreasing by 5%, indicating that the nanozyme is resistant to various ambient temperatures. 

Because the enzyme might be used in an actual aqueous system, the effect of ionic strength on its activity needs to be explored. In this context, Figure 5c compares the catalytic results of laccase and the Cu-MIM nanozyme when they are mixed with different NaCl concentrations. The catalytic activity of laccase decreases sharply with the increase in ion concentration, and at a 500 mM NaCl concentration, laccase was almost inactivated. This is because a high Na^+^ ion concentration can significantly affect the charge distribution and spatial structure of laccase, and the salting-out effect leads to a decrease in the solubility of laccase [51]. In addition, chloride ion is an inhibitor of laccase T2, T3 active site, which leads to laccase inactivation [52]. The catalytic performance of the Cu-MIM nanozyme is slightly enhanced with the increase in the NaCl concentration. We measured the Zeta potential of Cu-MIM, which was 12.2 mV in the absence of NaCl, and dropped to 9.4 mV with the addition of NaCl. This may be because the presence of NaCl facilitates the adsorption of 2,4-DP on the Cu-MIM nanozyme, thereby enhancing the catalytic performance [40].

To study the effect of organic solvent on the catalytic activity of the nanozyme, different amounts of ethanol were added into the reaction system. As shown in Figure 5d, the catalytic activity of laccase decreased linearly with the increase in ethanol, because ethanol itself can affect the structure of the natural enzyme, making the enzyme inactive. However, the catalytic activity of the Cu-MIM nanozyme is hardly affected by ethanol, and the catalytic activity is maintained at about 100% in various systems containing different amounts of ethanol, which fully indicates that ethanol has no effect on the structure of the nanozyme.

When enzymes are used in daily life, certain storage stability is required. We dissolved both laccase and the nanozyme in water and tested their catalytic activity for two weeks. As shown in Figure 5e, the activity of laccase gradually decreases in the first 8 days, and the activity decreased by 80% on the 8th day, basically in a state of inactivation. Cu-MIM nanozyme shows very excellent storage stability. Although the activity shows a gradual decrease in the 14 days, the proportion of decrease is very small, and the catalytic activity decreases by only 10% in 14 days. Meanwhile, the Cu-MIM nanozyme and laccase were dissolved in water for 100 days and their activities were tested on the first day and hundredth day. As shown in Figure 5f, after 100 days, laccase is unquestionably inactive while the Cu-MIM nanozyme retains 80% activity. These results show that the Cu-MIM nanozyme has excellent storage stability.

### 2.4. Degradation or Detection of Other Substances by the Cu-MIM Nanozyme

**Figure 6:**
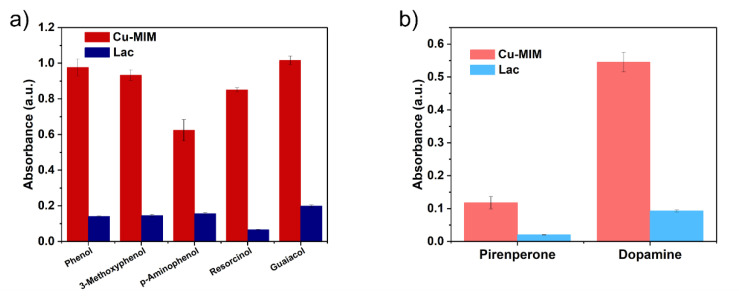
(**a**) Catalytic efficiency of Cu-MIM and laccase for different substrates and (**b**) detection capability of Cu-MIM and laccase for two biomolecules.

In order to verify the broadness of Cu-MIM substrates, we performed catalytic reactions on several phenolic pollutants such as 3-methoxyphenol, 4-aminophenol, phenol, resorcinol and guaiacol with 4-AP. We also tested two biomolecules such as epinephrine and dopamine, and absorbance values were measured at 485 nm and 290 nm, respectively. The results are shown in Figure 6. Cu-MIM nanozyme shows applicable activities to a variety of phenolic compounds, and the catalytic results are much higher than laccase. In addition, the Cu-MIM nanozyme also shows good catalytic abilities to epinephrine and dopamine, indicating its application in the detection of biomolecules.

### 2.5. Intelligent Detection of Phenol by the Cu-MIM Nanozyme

As a common toxic substance, it is very important to detect and degrade phenol conveniently and efficiently. As shown in Figure 6a, we have demonstrated that the Cu-MIM nanozyme has catalytic ability for phenol, then the kinetic parameters of the reaction are explored (Figure S9). As shown in Table 2, the *K*_m_ of the Cu-MIM nanozyme is lower than that of laccase and the *v*_max_ of Cu-MIM is 6.8 times higher than that of laccase, indicating that the Cu-MIM nanozyme has a better affinity for phenol and a faster reaction rate than laccase.

In order to determine the detection limit of the Cu-MIM nanozyme for phenol, different concentrations of phenol (0.5–4 μg/mL) were mixed with the catalyst (Figure 6a). The detection limits of laccase and the Cu-MIM nanozyme for phenol are 0.57 μg/mL and 0.19 μg/mL, respectively. The detection limit of the Cu-MIM nanozyme is 3 times lower than that of laccase, indicating that it has a more prominent detection ability.

**Figure 7:**
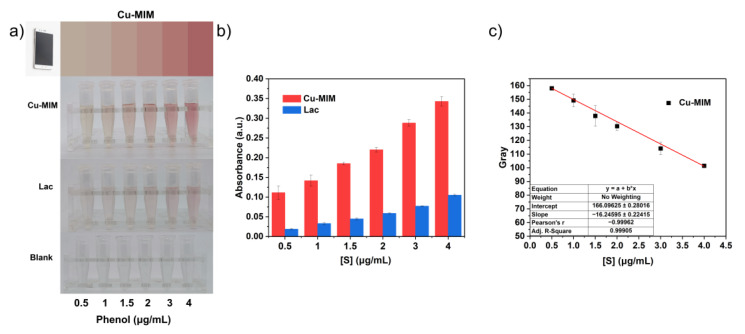
(**a**) Reactions of laccase and Cu-MIM with different concentrations of phenol and color standard card for the reaction of Cu-MIM with phenol (the blank group means there is no catalyst added); (**b**) Absorbance at 510 nm of the reaction products of laccase and Cu-MIM with different concentrations of phenol; (**c**) The correlation curve between color depth and phenol concentration is fitted by the RGB value of the standard color card.

There is a color change in the catalytic reaction of phenol with 4-AP, so we built a standard color detector for the rapid and efficient detection of phenol. Optical colorimetric detection of substances using smartphones is a new and convenient method, but generally this method is affected by device hardware and photographic quality. There may be some differences in the color of photos taken by different devices, which is unavoidable. Specifically, the quality of the photos taken can be affected by conditions such as light intensity, shooting distance, shooting angle, sample position, etc., which may cause color non-uniformity, distortion and distortion errors between different samples. In order to reduce the error, fixed shooting conditions and repeated experiments are necessary. We fixed the mobile phone directly above the sample at 20 cm, maintained a certain light intensity and photographed the sample at the same position, and repeated the experiment three times (Figure S10). We collected the color information of phenol catalyzed by Cu-MIM for 1 h with the Color Collect App, and converted it into a standard color card (Figure 7a). Then, we fit the equation reflecting the color depth according to the RGB value (Table S2) of the standard color card, as shown in Figure 7c. The color change process is fast during detection and the color change can be observed with the naked eye within tens of seconds after mixing the reaction system. Therefore, we established a fast, convenient and precise detection and quantification of phenol using the Cu-MIM nanozyme through a smartphone.

We investigated the anti-interference ability of Cu-MIM to detect phenol. As shown in Figure S11, L-cysteine, L-tyrosine, calcium chloride, magnesium chloride, potassium chloride and glucose were far less than phenol in terms of color change and absorbance. It showed that the method based on Cu-MIM for the detection of phenol had a certain anti-interference ability. Of course, this method also has certain shortcomings. Most phenolic compounds produce red reactions, so their detection specificity needs to be further optimized.

## 3. Experimental

### 3.1. Chemicals

2,4-dichlorophenol (2,4-DP), 4-aminoantipyrine (4-AP), phenol, 3-methoxylphenol, p-aminophenol, resorcinol, guaiacol, epinephrine and dopamine were from Macklin Biochemical Co., Ltd. (Shanghai, China). 2-(N-Morpholino) ethane sulfonic acid (MES) monohydrate, copper (II) chloride, imidazole, 1-methylimidazole, 1-ethylimidazole, 1-propylimidazole and 1-butylimidazole were from Aladdin Inc. (Shanghai, China). Laccase (Lac 1070 u/g) and Horseradish Peroxidase (HRP) were from Yuanye Biotechnology Co., Ltd. (Shanghai, China). All other chemicals, such as anhydrous ethanol, 2,2′-azino-bis (3-ethylbenzothiazoline-6-sulfonic acid) (ABTS) and aqueous hydrogen peroxide solution (H_2_O_2_, 30 wt%) were from Sinopharm Chemical Reagent Co., Ltd. (Shanghai, China). Milli-Q water was used to prepare the buffers and solutions. 

### 3.2. Synthesis of the Cu-Nanozymes

At room temperature, 0.5 mmol of ligand (i.e., imidazole, 1-methylimidazole, 1-ethylimidazole, 1-propylimidazole and 1-butylimidazole) and 0.5 mmol of CuCl_2_ were successively added to 5 mL of absolute ethanol, and magnetically stirred (300 r/min) for 1 h. After that, the reaction system was centrifuged at 11,672× *g* for 10 min, washed with ethanol 2–3 times and dried at 60 °C to obtain the products.

### 3.3. Characterizations

Scanning electron microscopy (SEM) images were recorded using a thermal field emission scanning electron microscope (FESEM, JSM-7001F, Hitachi High-technologies Co., Tokyo, Japan) at an acceleration voltage of 5 kV. All samples were sputter coated with platinum using an E1045 Pt coater (Hitachi High-Technologies Co., Tokyo, Japan) before SEM observation. The Fourier transform infrared spectroscopy (FTIR) was recorded by a Shimadzu tracer-100 infrared spectrophotometer (Shimadzu, Kyoto, Japan) using KBr disc technique, of which the frequency range was from 400 to 4000 cm^−1^. The ultraviolet–visible spectroscopy (UV-Vis) was carried out on a U3900 infrared spectrophotometer (Shimadzu, Kyoto, Japan). The XPS spectra was recorded using X-ray photoelectron spectroscopy (ESCALAB 250Xi, ThermoFischer, Waltham, MA, USA), which were divided peaks using Avantage. Inductively coupled plasma-atomic emission spectroscopy (ICP-AES) was performed on an Agilent 725ES (Agilent, Santa Clara, CA, USA). The Zeta potential measurements were measured using a Zeta potential analyzer Litesizer™500 (Anton Paar, Graz, Austria). The sample was prepared by dispersing 0.1 mg of the Cu-MIM nanozyme into 1 mL MES buffer (pH = 6.8) with 150 mM NaCl. X-ray diffraction (XRD) was performed on an X-ray diffractometer (Bruker, Germany) using Cu Kα radiation (λ = 1.5178 Å, 40 kV × 40 mA), and 2θ was scanned from 10° to 85° at 8°/min. The specific surface area was measured by N_2_ adsorption–desorption isotherms, which was conducted using a Micromeritics ASAP2460 machine.

### 3.4. Catalytic Activity Assays

The catalytic performance was measured by the chromogenic reaction of phenolic compounds with 4-AP. Specifically, 4-AP (1 mg/mL, 100 μL) and 2,4-DP (1 mg/mL, 100 μL) aqueous solutions were mixed with MES buffer (30 mM, pH 6.8, 700 μL). Then, the nanozymes or laccase solution (1 mg/mL, 100 μL) was added into the mixture at room temperature. After 20 min, the mixture was centrifuged at 11125× *g* for 2 min. The absorbance of the supernatant at 335 nm was measured. The other substrates (phenol, guaiacol, 3-methoxylphenol and o-phenylenediamine) were dissolved at 100 μg/mL in MES buffer (30 mM, pH 6.8) containing 100 μg/mL 4-AP and assayed in the same way. Epinephrine and dopamine reacted with laccase or Cu-MIM in the MES buffer (30 mM, pH 6.8) and absorbance was measured at 485 nm and 290 nm, respectively.

### 3.5. Determination of Catalytic Kinetic Parameters

Various concentrations of 2,4-DP (i.e., 1, 2, 4, 6, 8 and 10 μg/mL) were, respectively, reacted with Cu/GMP or laccase (0.1 mg/mL) and 4-AP (0.1 mg/mL) to measure the initial reaction rate. All reactions were carried out in MES buffer (30 mM, pH 6.8). The kinetic parameters (*K*_m_ and *v*_max_) were calculated using the Michaelis–Menten equation: 1/V_0_ = (*K*_m_/*v*_max_) (1/[S_0_] + 1/*v*_max_).

### 3.6. Assessment of Catalytic Stability

To study the effect of pH, Cu-MIM or laccase was incubated at different pH buffers (2–10) for 8 h before the activity test. The relative activity was compared with the activity at pH 7. To study the effect of temperature, Cu-MIM or laccase was stored at various temperatures (20–90 °C) for 30 min before activity measurement, and the activity at 30 °C was taken as a reference. To study the effect of ionic strength, different concentrations of NaCl (0, 150, 300 and 500 mM) were added into the reaction and the activity at 0 mM was taken as a reference. To study the effect of organic solvent, different amounts of ethanol (0, 25%, 50%, 75% and 100% *v*/*v*) were mixed with the reactants, and the activity at 0% of ethanol was taken as a reference. To study the storage stability, laccase or Cu-MIM was stored in aqueous solution for 14 days, and the catalytic activity was measured once a day. The first day was taken as a reference. In addition, Cu-MIM or laccase was stored in DI water for 100 days and their activities were tested on the first day and hundredth day. For all the studies, the absorbance of the supernatant was measured at 335 nm after 20 min of reaction. 

### 3.7. Intelligent Detection of Phenol

To determine the detection limit of phenol, different concentrations of phenol (0.5, 1, 1.5, 2, 3 and 4 μg/mL) were reacted with Cu-MIM (0.1 mg/mL) or laccase (0.1 mg/mL) for 1 h in MES buffer, and the absorbance values at 510 nm were measured. The limit of detection was calculated by 3σ/b, where σ is the standard deviation of the blank signals and b is the slope of the regression line.

Take pictures of phenol at each concentration with the Color Collect App on a smartphone, and convert the color to a standard color chart based on the color in the picture. The RGB values in the standard color chart can be calculated according to the formula (g = R × 0.299 + G × 0.587 + B × 0.114) to reflect the gray values of the color depth. Then, a concentration-related standard curve is established based on the gray values for the intelligent detection of phenol. 

In order to study the anti-interference ability of Cu-MIM to detect phenol, common interfering substances such as L-cysteine, L-tyrosine, calcium chloride, magnesium chloride, potassium chloride and glucose (0.1 mg/mL) were mixed with Cu-MIM (0.1 mg/mL) and 4-AP (0.1 mg/mL) in 1 mL MES buffer for 20 min, and the absorbance at 510 nm was measured.

## 4. Conclusions

In summary, we synthesized a series of nanozymes with laccase-like activity from cheap imidazole derivatives and confirmed that the Cu-MIM nanozyme with a C1 chain in the imidazole ring was the best. Compared with laccase, the Cu-MIM nanozyme exhibited stronger catalytic activity and a faster reaction rate. Additionally, the Cu-MIM nanozyme exhibited excellent stability under various harsh conditions, showed good activity at high temperature, high salt, ethanol and extreme pH and remained highly active after prolonged storage. In addition, it showed good catalytic abilities to a variety of phenolic pollutants and also had a certain ability to detect biomolecules epinephrine and dopamine. Despite the excellent properties exhibited by the Cu-MIM nanozyme, there are still some questions that need to be considered in future research, including whether the activity of the Cu-MIM nanozyme is disturbed by organic pollutants in wastewater, whether the Cu-MIM nanozyme is potentially toxic in water and the condition optimization for large-scale preparation of the Cu-MIM nanozyme. 

Finally, relying on the strong catalytic activity of the Cu-MIM nanozyme, we designed a smart phenol detector that can quantify the phenol concentration through color change. We believe that this nanozyme with laccase-like activity has great application potential in biomimetic catalysis, environmental remediation and biosensing.

## Figures and Tables

**Table 1:** Kinetic parameters of the reaction of 2,4-DP and 4-AP catalyzed by laccase and nanozymes.

Catalyst	Substrate	*K*_m_ (mol·L^−1^)	*V*_max_ (mol·L^−1^·s^−1^)	Ref.
Cu/GMP	2,4-DP	0.00059	0.000013	[5]
CH-Cu	2,4-DP	0.00042	0.000122	[40]
Cu_2_O	2,4-DP	0.000203	10^−7^	[34]
I-Cu	2,4-DP	0.000172	4.1 × 10^−7^	[41]
Laccase	2,4-DP	0.0000116	0.0000578	This work
Cu-MIM	2,4-DP	0.0000448	0.000491	This work

**Table 2:** Kinetic parameters of the reaction of phenol and 4-AP catalyzed by Cu-MIM or laccase.

Catalyst	*K*_m_ (mol·L^−1^)	*V*_max_ (mol·L^−1^·s^−1^)
Cu-MIM	0.0000505	0.000595
Laccase	0.000087	0.00008

## Data Availability

Not applicable.

## Supplementary Materials

The following supporting information can be downloaded at: https://www.mdpi.com/article/10.3390/molecules27154712/s1, Figure S1. SEM images of nanozymes: (a) Cu-IM, (b) Cu-EIM, (c) Cu-PIM, and (d) Cu-BIM. Figure S2. XRD patterns of nanozymes prepared in the current work. Figure S3. FTIR spectrum: (a) Cu-IM, (b) Cu-EIM, (c) Cu-PIM, and (d) Cu-BIM. Figure S4. (a)XPS fully scanned spectrum of the Cu-MIM nanozyme, (b) N_2_ adsorption-desorption isotherms of different samples. Figure S5. N 1s XPS spectrum: (a) Cu-IM, (b) Cu-MIM (c) Cu-EIM, (d) Cu-PIM, and (e) Cu-BIM. Figure S6. Wavelengths of Cu-MIM catalyzed 2, 4-DP and 4-AP reaction (a), product absorbance versus time catalyzed by laccase or Cu-MIM (b), and comparison of reaction catalyzed by Cu-MIM or CuCl_2_ for 20 min (c), (d) comparison of reaction catalyzed by Cu-MIM or 1-Methylimidazole. Figure S7. Molecular structures of five phenolic compounds. Figure S8. Lineweaver-Burk plot for Cu-MIM nanozyme and laccase oxidizing 2, 4-DP at room temperature. Figure S9. Lineweaver-Burk plot for Cu-MIM nanozyme and laccase oxidizing phenol at room temperature. Figure S10. Photographs of the reaction of different concentrations of phenol and Cu-MIM nanozymes. Figure S11. The selectivity of Cu-MIM nanozyme towards phenol and other potential interferential substances. Table S1. The FT-IR spectral summary of Cu-MIM nanozyme and 1-methylimidazole dipeptide. Table S2. The RGB value corresponding to the standard color chart of each phenol concentration.

## Sample Availability

Samples of the compounds are not available from the authors.

## References

[1] Wolfenden R., Snider M.J. (2001). The Depth of Chemical Time and the Power of Enzymes as Catalysts. Acc. Chem. Res..

[2] Garcia-Viloca M., Gao J., Karplus M., Donald G.T. (2004). How Enzymes Work: Analysis by Modern Rate Theory and Computer Simulations. Science.

[3] Lin Y., Ren J., Qu X. (2014). Catalytically Active Nanomaterials: A Promising Candidate for Artificial Enzymes. Acc. Chem. Res..

[4] Wu Y., Jiao L., Luo X., Xu W., Wei X., Wang H., Yan H., Gu W., Xu B.Z., Du D. (2019). Oxidase-Like Fe-N-C Single-Atom Nanozymes for the Detection of Acetylcholinesterase Activity. Small.

[5] Liang H., Lin F., Zhang Z., Liu B., Jiang S., Yuan Q., Liu J. (2017). Multicopper Laccase Mimicking Nanozymes with Nucleotides as Ligands. ACS Appl. Mater. Interfaces.

[6] Liang M., Yan X. (2019). Nanozymes: From New Concepts, Mechanisms, and Standards to Applications. Acc. Chem. Res..

[7] Wei H., Wang E. (2013). Nanomaterials with enzyme-like characteristics (nanozymes): Next-generation artificial enzymes. Chem. Soc. Rev..

[8] Gao J., Zhao B., Wang M., Serrano M.A.C., Zhuang J., Ray M., Rotello V.M., Vachet R.W., Thayumanavan S. (2018). Supramolecular Assemblies for Transporting Proteins Across an Immiscible Solvent Interface. J. Am. Chem. Soc..

[9] Gao J., Le S., Thayumanavan S. (2021). Enzyme Catalysis in Non-Native Environment with Unnatural Selectivity Using Polymeric Nanoreactors. Angew. Chem. Int. Ed..

[10] Feng Z., Zhang T., Wang H., Xu B. (2017). Supramolecular catalysis and dynamic assemblies for medicine. Chem. Soc. Rev..

[11] Sharma A., Gupta G., Ahmad T., Mansoor S., Kaur B. (2021). Enzyme Engineering: Current Trends and Future Perspectives. Food Rev. Int..

[12] Nguyen H.H., Kim M. (2017). An Overview of Techniques in Enzyme Immobilization. Appl. Sci. Converg. Technol..

[13] Gao L., Zhuang J., Nie L., Zhang J., Zhang Y., Gu N., Wang T., Feng J., Yang D., Perrett S. (2007). Intrinsic peroxidase-like activity of ferromagnetic nanoparticles. Nat. Nanotechnol..

[14] Wu J., Wang X., Wang Q., Lou Z., Li S., Zhu Y., Qin L., Wei H. (2019). Nanomaterials with enzyme-like characteristics (nanozymes): Next-generation artificial enzymes (II). Chem. Soc. Rev..

[15] Giljohann D.A., Seferos D.S., Daniel W.L., Massich M.D., Patel P.C., Mirkin C.A. (2010). Gold Nanoparticles for Biology and Medicine. Angew. Chem. Int. Ed..

[16] Lin Y., Li Z., Chen Z., Ren J., Qu X. (2013). Mesoporous silica-encapsulated gold nanoparticles as artificial enzymes for self-activated cascade catalysis. Biomaterials.

[17] Feng L., Zhang L., Chu S., Zhang S., Chen X., Du Z., Gong Y., Wang H. (2022). Controllable doping of Fe atoms into MoS_2_ nanosheets towards peroxidase-like nanozyme with enhanced catalysis for colorimetric analysis of glucose. Appl. Surf. Sci..

[18] Korsvik C., Patil S., Seal S., Self W.T. (2007). Superoxide dismutase mimetic properties exhibited by vacancy engineered ceria nanoparticles. Chem. Commun..

[19] Frey N.A., Peng S., Cheng K., Sun S. (2009). Magnetic nanoparticles: Synthesis, functionalization, and applications in bioimaging and magnetic energy storage. Chem. Soc. Rev..

[20] Hu S., Zhang W., Li N., Chang Q., Yang J. (2021). Integrating biphase γ- and α-Fe_2_O_3_ with carbon dots as a synergistic nanozyme with easy recycle and high catalytic activity. Appl. Surf. Sci..

[21] Zhang Q., He X., Han A., Tu Q., Fang G., Liu J., Wang S., Li H. (2016). Artificial hydrolase based on carbon nanotubes conjugated with peptides. Nanoscale.

[22] Song Y., Qu K., Zhao C., Ren J., Qu X. (2010). Graphene Oxide: Intrinsic Peroxidase Catalytic Activity and Its Application to Glucose Detection. Adv. Mater..

[23] He B., Feng M., Chen X., Sun J. (2021). Multidimensional (0D-3D) functional nanocarbon: Promising material to strengthen the photocatalytic activity of graphitic carbon nitride. Green Energy Environ..

[24] Li P., Klet R.C., Moon S.-Y., Wang T.C., Deria P., Peters A.W., Klahr B.M., Park H.-J., Al-Juaid S.S., Hupp J.T. (2015). Synthesis of nanocrystals of Zr-based metal–organic frameworks with csq-net: Significant enhancement in the degradation of a nerve agent simulant. Chem. Commun..

[25] Nunes P., Gomes A.C., Pillinger M., Gonçalves I.S., Abrantes M. (2015). Promotion of phosphoester hydrolysis by the ZrIV-based metal-organic framework UiO-67. Microporous Mesoporous Mater..

[26] Ji J., Ko S.Y., Choi K.M., Kwon Y. (2021). Hydrogen peroxide sensor using the biomimetic structure of peroxidase including a metal organic framework. Appl. Surf. Sci..

[27] Huang Y., Ren J., Qu X. (2019). Nanozymes: Classification, Catalytic Mechanisms, Activity Regulation, and Applications. Chem. Rev..

[28] Wang H., Wan K., Shi X. (2019). Recent Advances in Nanozyme Research. Adv. Mater..

[29] Lainé J., Foucaud Y., Bonilla-Petriciolet A., Badawi M. (2022). Molecular picture of the adsorption of phenol, toluene, carbon dioxide and water on kaolinite basal surfaces. Appl. Surf. Sci..

[30] Nair C.I., Jayachandran K., Shashidhar S. (2008). Biodegradation of phenol. Afr. J. Biotechnol..

[31] Bruce R.M., Santodonato J., Neal M.W. (1987). Summary Review of the Health Effects Associated with Phenol. Toxicol. Ind. Health.

[32] Zhong N., Yuan J., Luo Y., Zhao M., Luo B., Liao Q., Chang H., Zhong D., Rittmann B.E. (2021). Intimately coupling photocatalysis with phenolics biodegradation and photosynthesis. Chem. Eng. J..

[33] Li D., Cheng Y., Zuo H., Zhang W., Pan G., Fu Y., Wei Q. (2021). Dual-functional biocatalytic membrane containing laccase-embedded metal-organic frameworks for detection and degradation of phenolic pollutant. J. Colloid Interface Sci..

[34] Maity T., Jain S., Solra M., Barman S., Rana S. (2022). Robust and Reusable Laccase Mimetic Copper Oxide Nanozyme for Phenolic Oxidation and Biosensing. ACS Sustain. Chem. Eng..

[35] Shu R., Li R., Lin B., Luo B., Tian Z. (2020). High dispersed Ru/SiO_2_-ZrO_2_ catalyst prepared by polyol reduction method and its catalytic applications in the hydrodeoxygenation of phenolic compounds and pyrolysis lignin-oil. Fuel.

[36] Catherine H.N., Ou M.-H., Manu B., Shih Y.H. (2018). Adsorption mechanism of emerging and conventional phenolic compounds on graphene oxide nanoflakes in water. Sci. Total Environ..

[37] Su J., Fu J., Wang Q., Silva C., Cavaco-Paulo A. (2018). Laccase: A green catalyst for the biosynthesis of poly-phenols. Crit. Rev. Biotechnol..

[38] Riva S. (2006). Laccases: Blue enzymes for green chemistry. Trends Biotechnol..

[39] Zhang S., Lin F., Yuan Q., Liu J., Li Y., Liang H. (2020). Robust magnetic laccase-mimicking nanozyme for oxidizing o-phenylenediamine and removing phenolic pollutants. J. Environ. Sci..

[40] Wang J., Huang R., Qi W., Su R., Binks B.P., He Z. (2019). Construction of a bioinspired laccase-mimicking nanozyme for the degradation and detection of phenolic pollutants. Appl. Catal. B.

[41] Wang J., Huang R., Qi W., Su R., He Z. (2022). Construction of biomimetic nanozyme with high laccase- and catecholase-like activity for oxidation and detection of phenolic compounds. J. Hazard. Mater..

[42] Quintanar L., Yoon J., Aznar C.P., Palmer A.E., Andersson K.K., Britt R.D., Solomon E.I. (2005). Spectroscopic and Electronic Structure Studies of the Trinuclear Cu Cluster Active Site of the Multicopper Oxidase Laccase:  Nature of Its Coordination Unsaturation. J. Am. Chem. Soc..

[43] Zhu X.-W., Luo D., Zhou X.-P., Li D. (2022). Imidazole-based metal-organic cages: Synthesis, structures, and functions. Coord. Chem. Rev..

[44] Platzman I., Brener R., Haick H., Tannenbaum R. (2008). Oxidation of Polycrystalline Copper Thin Films at Ambient Conditions. J. Phys. Chem. C.

[45] Lee W.-J. (2003). Inhibiting effects of imidazole on copper corrosion in 1 M HNO_3_ solution. Mater. Sci. Eng. A..

[46] Liu P., Hensen E.J.M. (2013). Highly Efficient and Robust Au/MgCuCr2O4 Catalyst for Gas-Phase Oxidation of Ethanol to Acetaldehyde. J. Am. Chem. Soc..

[47] Mathew J.P., Srinivasan M. (1995). Photoelectron spectroscopy (XPS) studies on some palladium catalysts. Eur. Polym. J..

[48] Wu J., Wang Z., Jin X., Zhang S., Li T., Zhang Y., Xing H., Yu Y., Zhang H., Gao X. (2021). Hammett Relationship in Oxidase-Mimicking Metal–Organic Frameworks Revealed through a Protein-Engineering-Inspired Strategy. Adv. Mater..

[49] Lu L., Zhao M., Wang Y. (2007). Immobilization of laccase by alginate-chitosan microcapsules and its use in dye decolorization. World J. Microbiol. Biotechnol..

[50] Dayaram P., Dasgupta D. (2008). Decolorisation of synthetic dyes and textile wastewater using Polyporus rubidus. J. Environ. Biol..

[51] Arakawa T., Timasheff S.N. (1982). Preferential interactions of proteins with salts in concentrated solutions. Biochemistry.

[52] Xu F. (1996). Oxidation of phenols, anilines, and benzenethiols by fungal laccases: Correlation between activity and redox potentials as well as halide inhibition. Biochemistry.

