# Supplementary material for: Facile Fabrication of 1-Methylimidazole/Cu Nanozyme with Enhanced Laccase Activity for Fast Degradation and Sensitive Detection of Phenol Compounds
Source: Molecules. 2022 Jul 23;27(15):4712. doi: 10.3390/molecules27154712 (PMC9331362; doi:10.3390/molecules27154712)
Supplement: Supplementary file 1 [file molecules-27-04712-s001.zip › molecules-1800946-supplementary.pdf]

Supporting Information

Facile fabrication of 1-methylimidazole/Cu nanozyme with enhanced laccase activity for fast degradation and sensitive detection of phenol compounds

Yu Lei [a], Bin He [a], Shujun Huang [a], Xinyan Chen [a], Jian Sun [a,b,*]

[a] Key Laboratory of Molecular Medicine and Biotherapy in the Ministry of Industry and Information Technology, School of Life Science, Beijing Institute of Technology, Beijing 100081, P.R. China

[b] Advanced Research Institute of Multidisciplinary Science, Beijing Institute of Technology, Beijing 100081, P.R. China

* Corresponding author. *E-mail address*: jiansun@bit.edu.cn

Number of pages: 8 pages

Number of Figures: 11

Number of Tables: 2

Contents:

Figure S1. SEM images of nanozymes: (a) Cu-IM, (b) Cu-EIM, (c) Cu-PIM, and (d) Cu-BIM.

Figure S2. XRD patterns of nanozymes prepared in the current work.

Figure S3. FTIR spectrum: (a) Cu-IM, (b) Cu-EIM, (c) Cu-PIM, and (d) Cu-BIM.

Figure S4. (a)XPS fully scanned spectrum of the Cu-MIM nanozyme, (b) N2 adsorption-desorption isotherms of different samples.

Figure S5. N 1s XPS spectrum: (a) Cu-IM, (b) Cu-MIM (c) Cu-EIM, (d) Cu-PIM, and (e) Cu-BIM.

Figure S6. Wavelengths of Cu-MIM catalyzed 2, 4-DP and 4-AP reaction (a), product absorbance versus time catalyzed by laccase or Cu-MIM (b), and comparison of reaction catalyzed by Cu-MIM or $CuCl_2$ for 20 min (c), (d) comparison of reaction catalyzed by Cu-MIM or 1-Methylimidazole.

Figure S7. Molecular structures of five phenolic compounds.

Figure S8. Lineweaver-Burk plot for Cu-MIM nanozyme and laccase oxidizing 2, 4-DP at room temperature.

Figure S9. Lineweaver-Burk plot for Cu-MIM nanozyme and laccase oxidizing phenol at room temperature.

Figure S10. Photographs of the reaction of different concentrations of phenol and Cu-MIM nanozymes.

Figure S11. The selectivity of Cu-MIM nanozyme towards phenol and other potential interferential substances.

Table S1. The FT-IR spectral summary of Cu-MIM nanozyme and 1-methylimidazole dipeptide.

Table S2. The RGB value corresponding to the standard color chart of each phenol concentration.

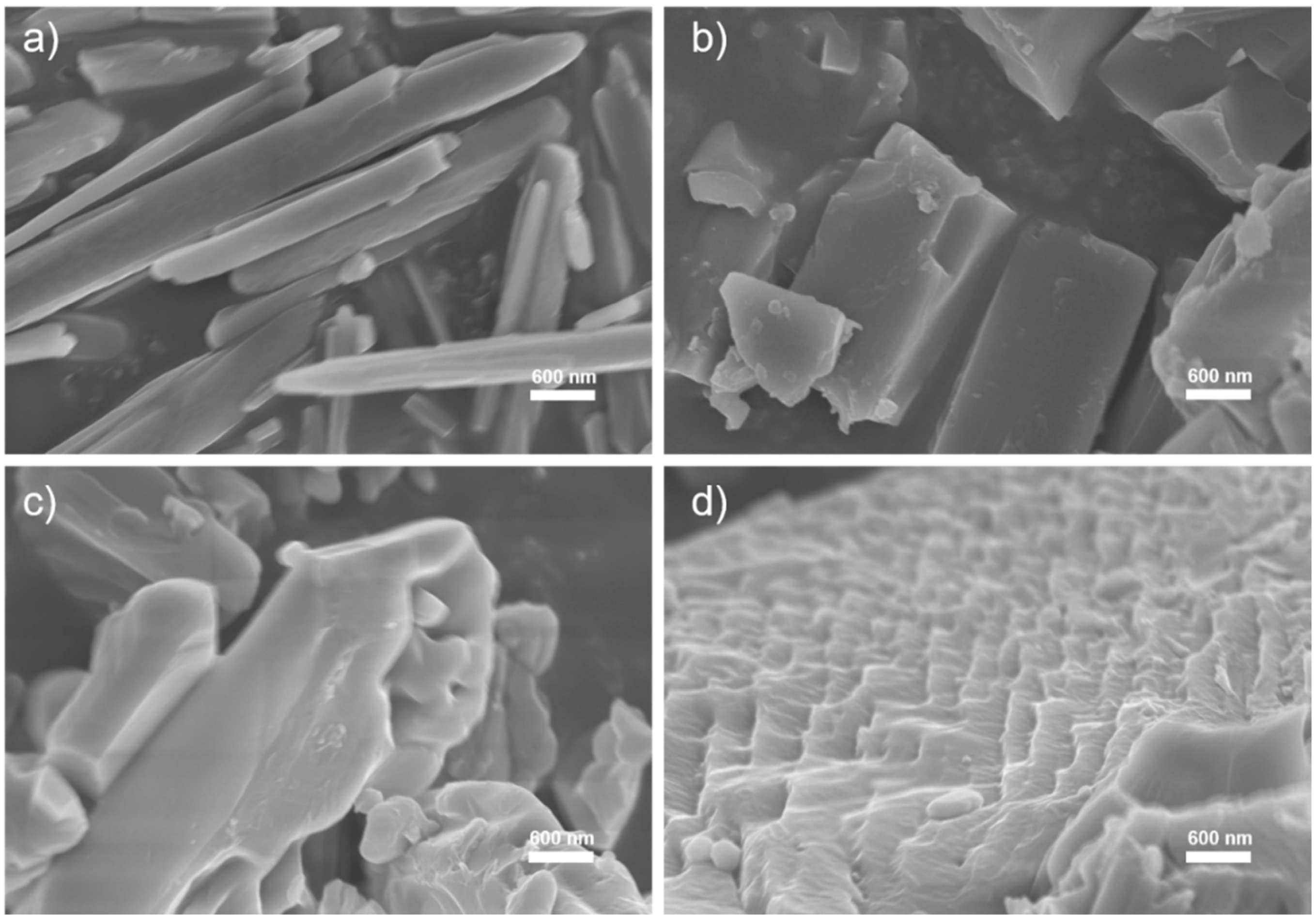

Figure S1. SEM images of nanozymes: (a) Cu-IM, (b) Cu-EIM, (c) Cu-PIM, and (d) Cu-BIM.

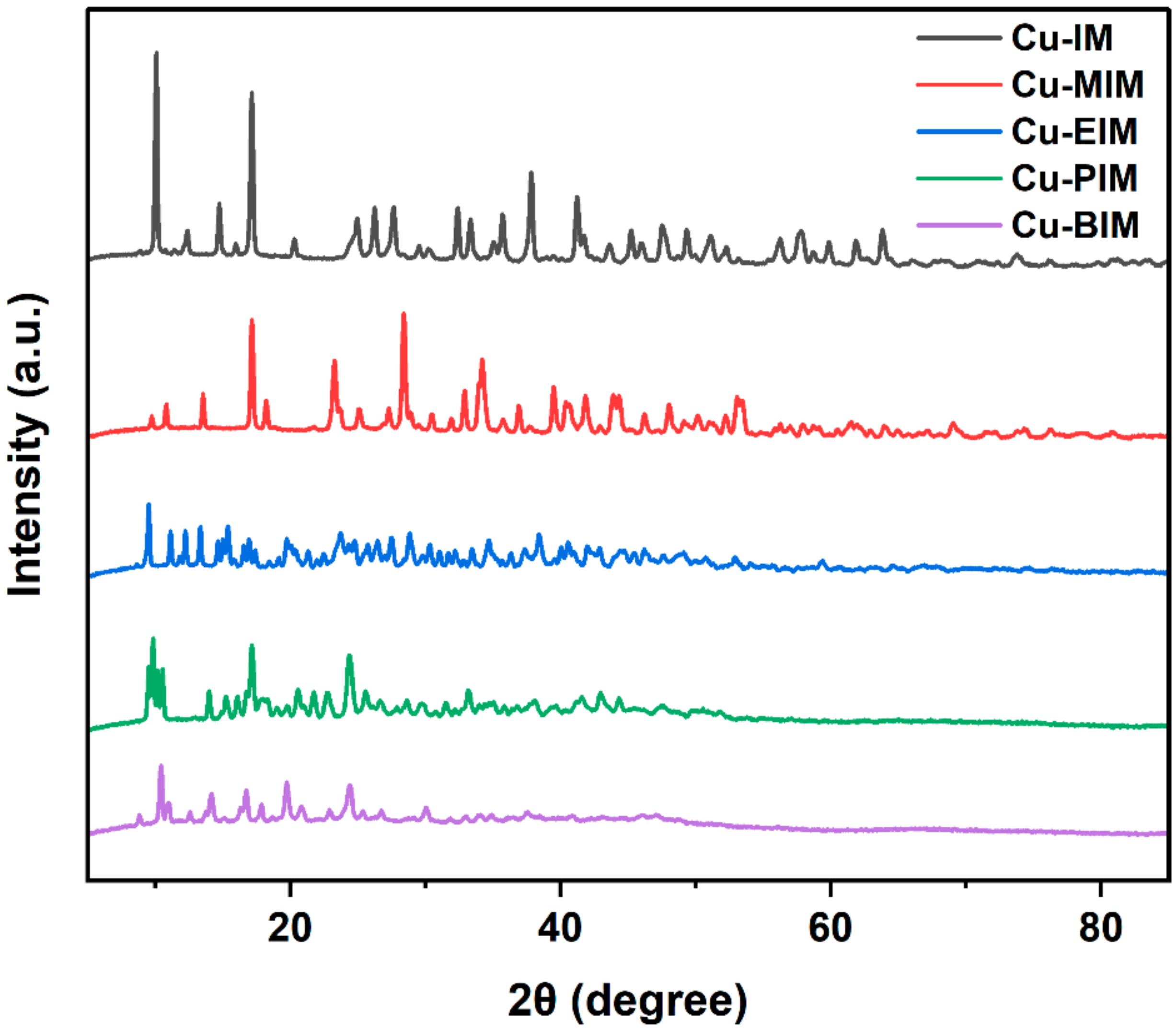

Figure S2. XRD patterns of nanozymes prepared in the current work.

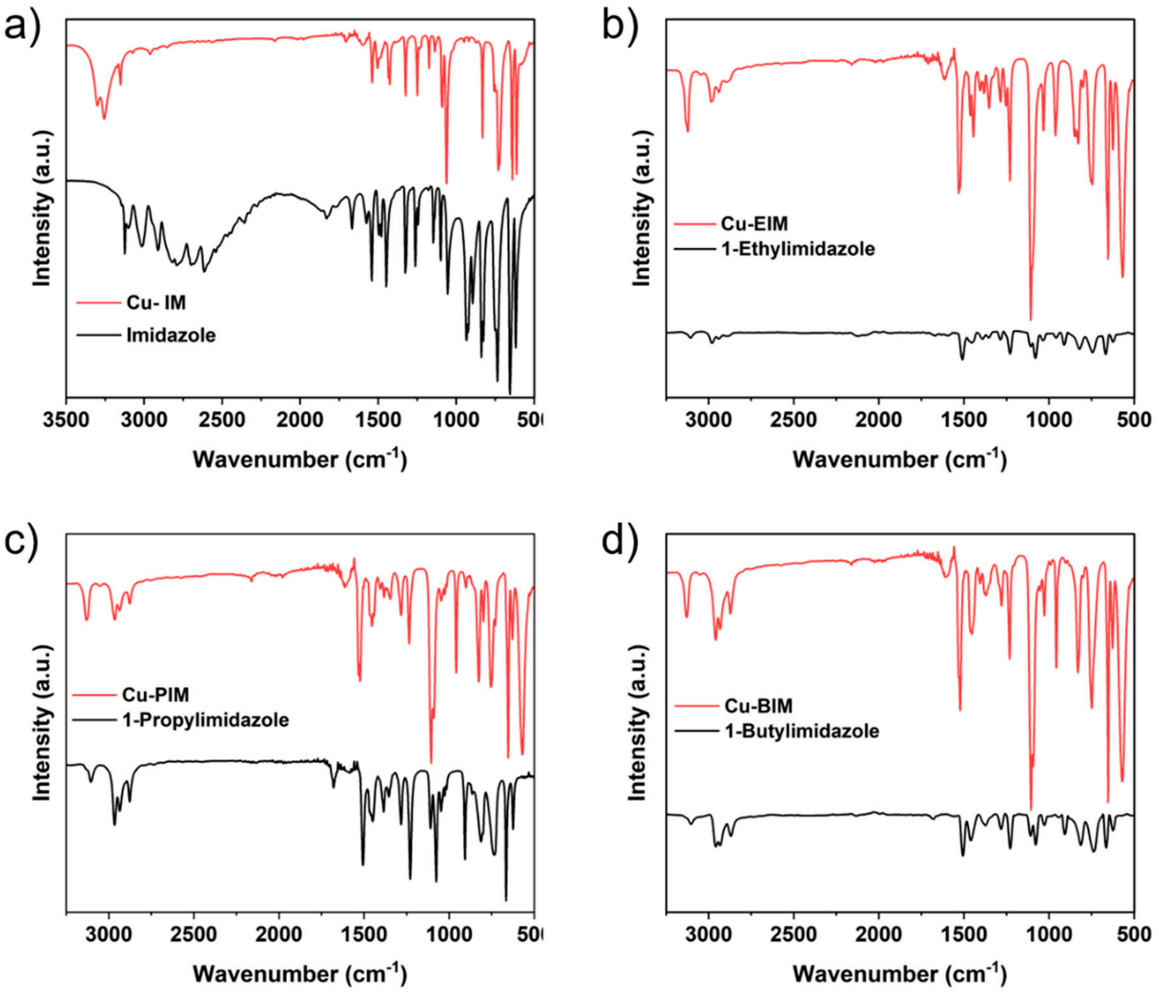

Figure S3. FTIR spectrum: (a) Cu-IM, (b) Cu-EIM, (c) Cu-PIM, and (d) Cu-BIM.

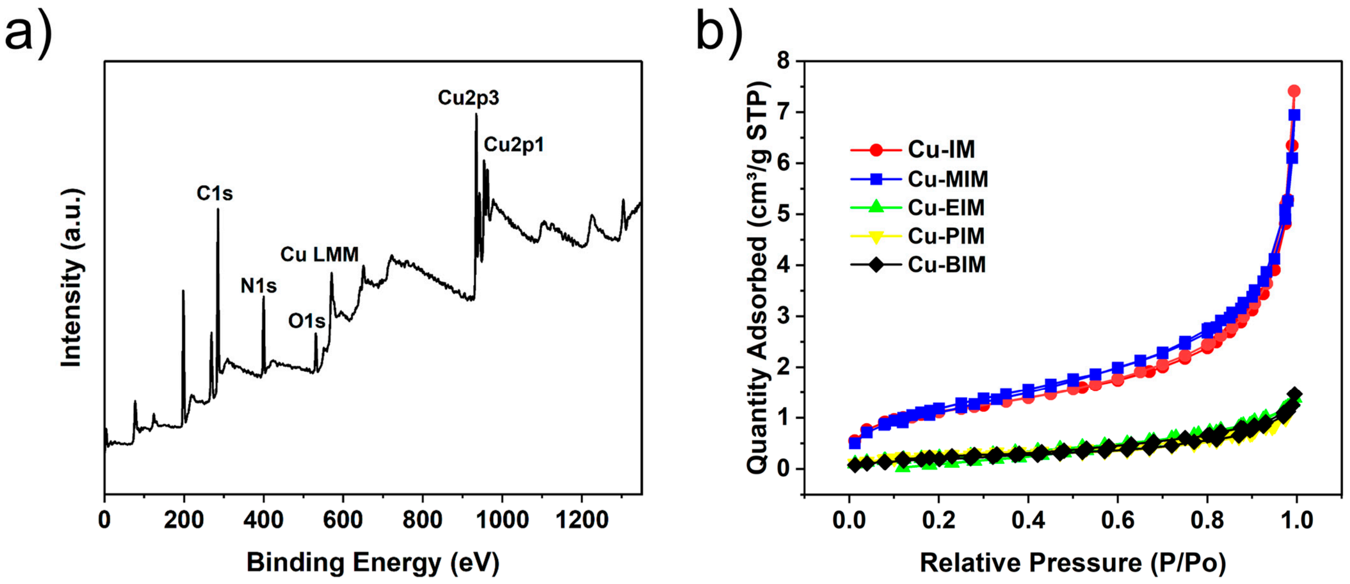

Figure S4. (a)XPS fully scanned spectrum of the Cu-MIM nanozyme, (b) N2 adsorption-desorption isotherms of different samples.

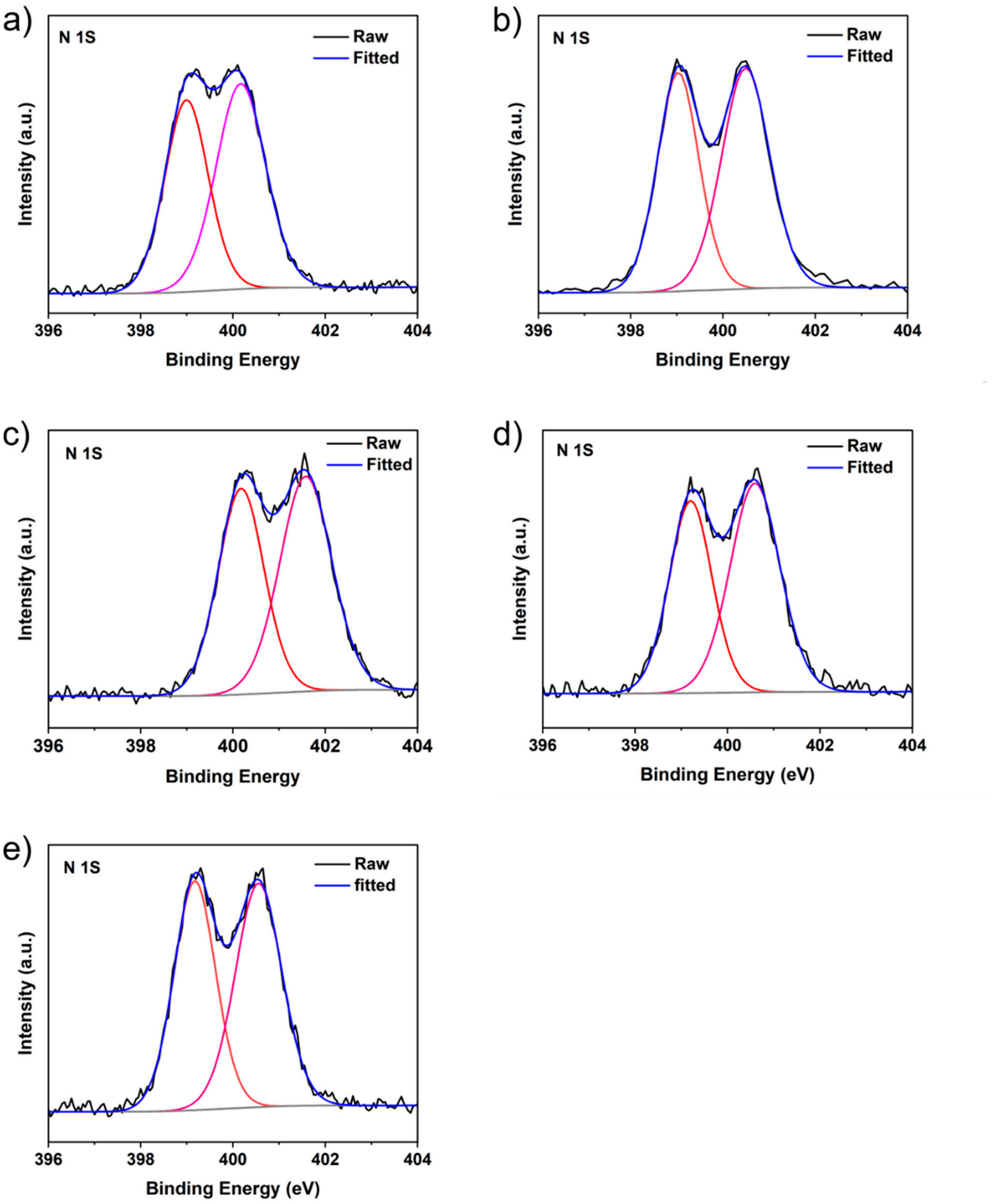

Figure S5. N 1s XPS spectrum: (a) Cu-IM, (b) Cu-MIM (c) Cu-EIM, (d) Cu-PIM, and (e) Cu-BIM.

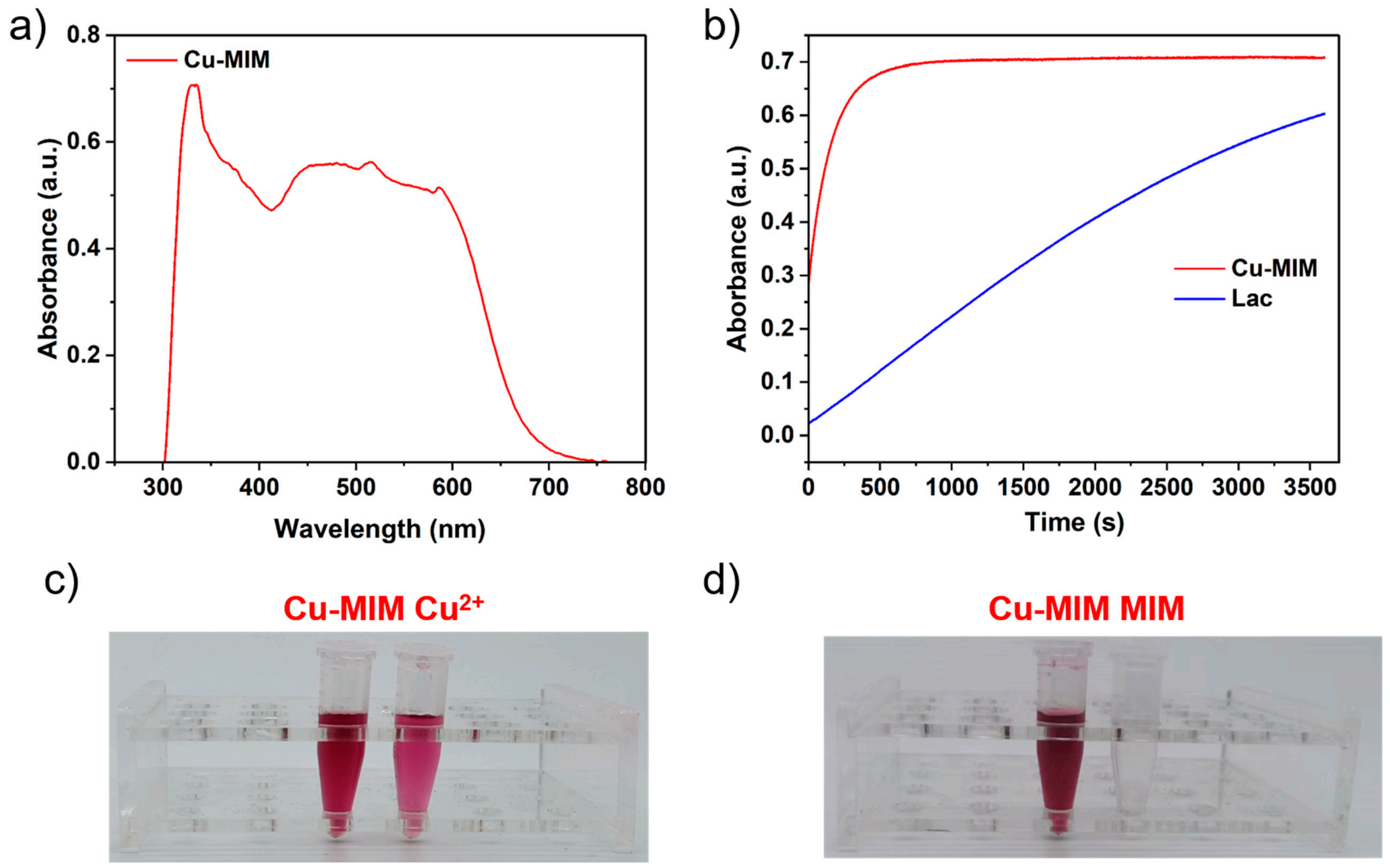

Figure S6. Wavelengths of Cu-MIM catalyzed 2, 4-DP and 4-AP reaction (a), product absorbance versus time catalyzed by laccase or Cu-MIM (b), and comparison of reaction catalyzed by Cu-MIM or $CuCl_2$ for 20 min (c), (d) comparison of reaction catalyzed by Cu-MIM or 1-Methylimidazole.

phenol 3-methoxylphenol p-aminophenol resorcinol guaiacol

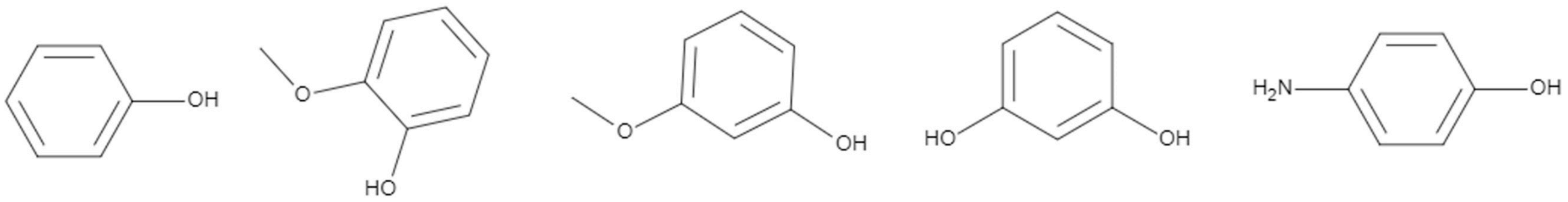

Figure S7. Molecular structures of five phenolic compounds.

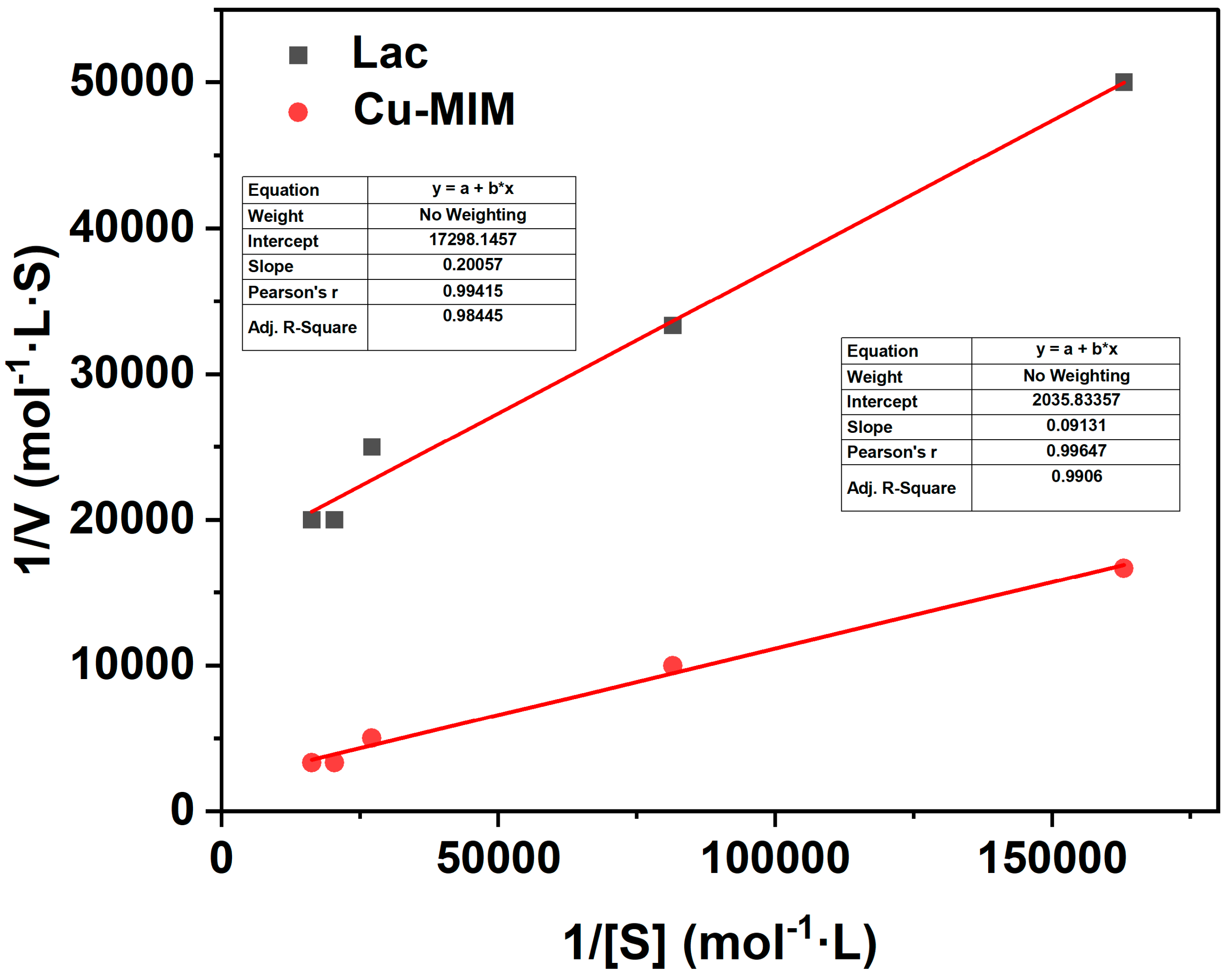

Figure S8. Lineweaver-Burk plot for Cu-MIM nanozyme and laccase oxidizing 2, 4-DP at room temperature.

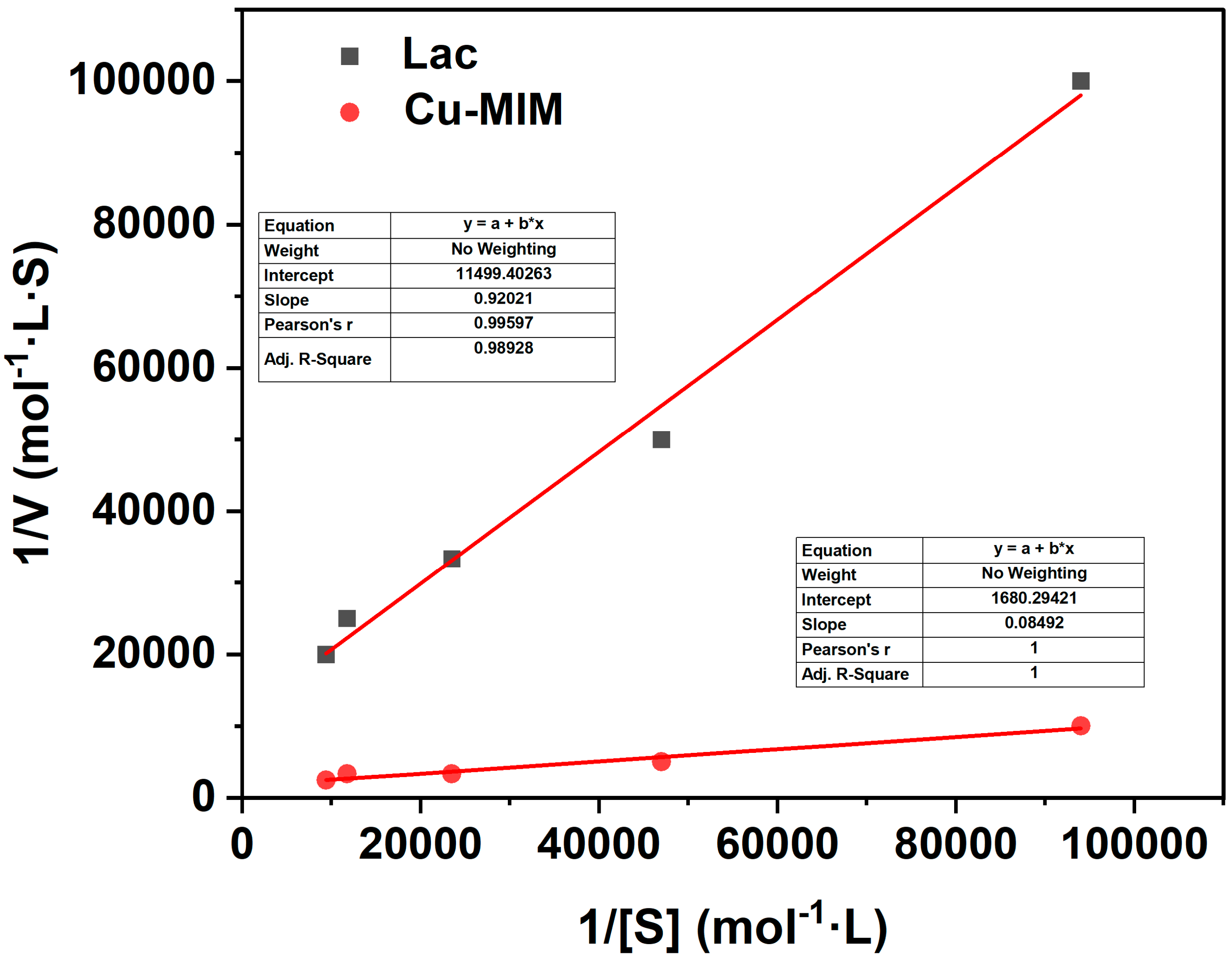

Figure S9. Lineweaver-Burk plot for Cu-MIM nanozyme and laccase oxidizing phenol at room temperature.

Table S1. FT-IR spectra of Cu-MIM nanozyme and 1-methylimidazole dipeptide.

**Chemical bond**	**Cu-MIM nanozyme/cm^{-1}**	**1-Methylimidazole/cm^{-1}**
ν(-C=C-H)	3129	3107
ν(-CH_3)	3074	2953
ν(C=N)	1622	1679
ν(C=C)	1598	1590
ν(N-C)	2927	2883
δ(C-H)	670	665

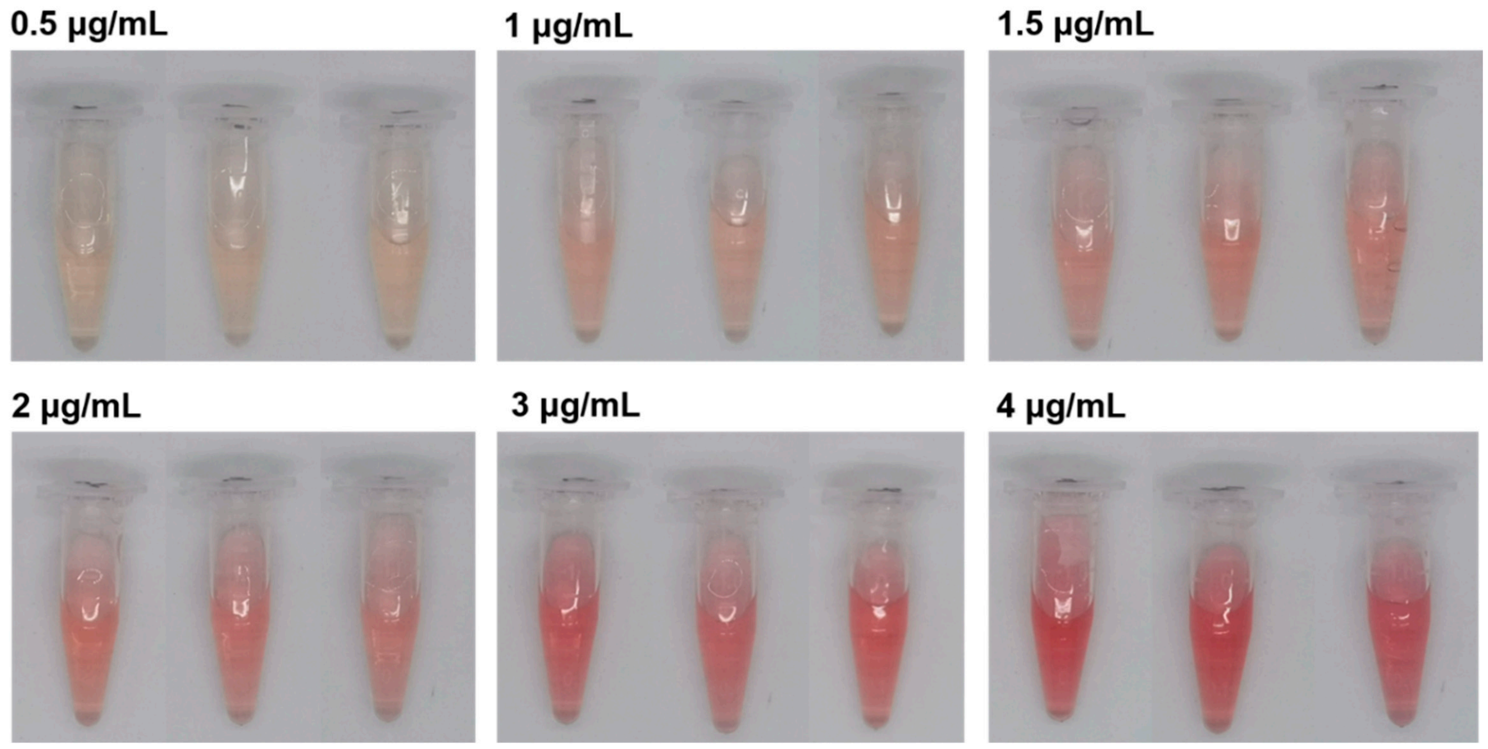

Figure S10. Photographs of the reaction of different concentrations of phenol and Cu-MIM nanozymes.

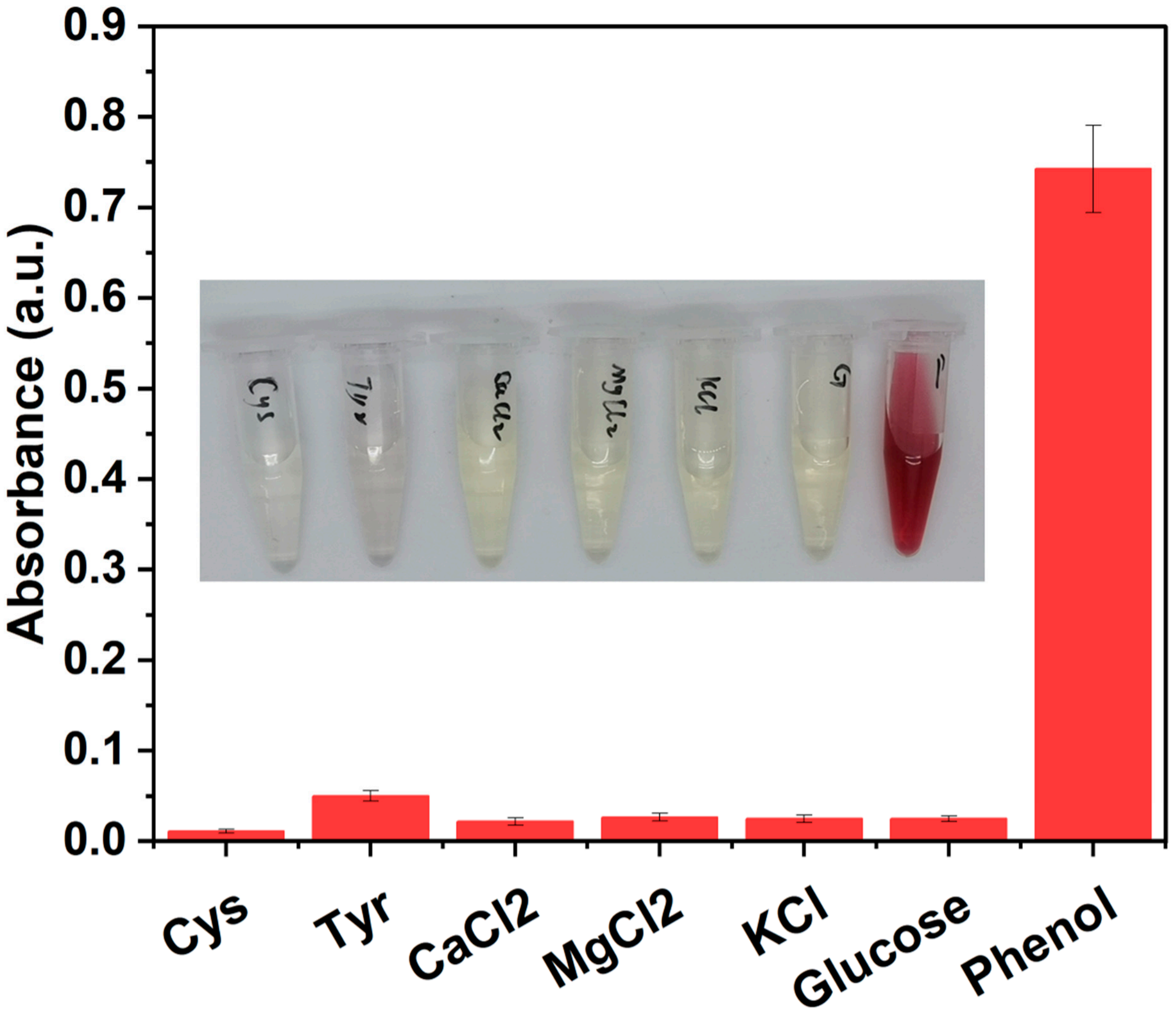

Figure S11. The selectivity of Cu-MIM nanozyme towards phenol and other potential interferential substances.

Table S2. The RGB values corresponding to the standard color chart of each phenol concentration.

[S] (μg/mL)	R1	G1	B1	R2	G2	B2	R3	G3	B3	g1	g2	g3	Mean	Standard Deviation	CV
4	154	79	86	157	75	81	158	77	83	102.223	100.202	101.903	101.4427	1.0863	0.01071
3	165	99	101	162	87	91	168	90	90	118.962	109.881	113.322	114.055	4.58466	0.0402
2	168	120	116	170	112	110	171	111	103	133.896	129.114	128.028	130.346	3.12197	0.02395
1.5	174	135	130	165	118	112	168	124	115	146.091	131.369	136.13	137.8633	7.5125	0.05449
1	173	146	137	174	142	131	168	136	123	153.047	150.314	144.086	149.149	4.59269	0.03079
0.5	169	155	142	173	154	139	175	154	137	157.704	157.971	158.341	158.0053	0.31988	0.00202
